# A Tension‐Controlled Arthroscopic Technique for Fixation of a Glenolabral Articular Disruption Lesion in an Overhead Athlete

**DOI:** 10.1002/atn2.70294

**Published:** 2026-10-23

**Authors:** Nathan Farner, Nicholas Zingas, Daria Bradley, Shariff K. Bishai

**Affiliations:** ^1^ Department of Orthopedics Mclaren Oakland Hospital Pontiac Michigan U.S.A.; ^2^ Department of Orthopedics Henry Ford Macomb Hospital Clinton Township Michigan U.S.A.; ^3^ Detroit Orthopaedic Institute PLLC Troy Michigan U.S.A.; ^4^ Michigan State University College of Osteopathic Medicine East Lansing Michigan U.S.A.; ^5^ Oakland University William Beaumont School of Medicine Rochester, New York U.S.A.; ^6^ Wayne State University School of Medicine Detroit Michigan U.S.A.; ^7^ Detroit Medical Center Sports Medicine Fellowship Detroit Michigan U.S.A

## Abstract

Glenolabral articular disruption lesions are rare post‐traumatic injuries that occur after shoulder instability events particularly in young, high‐demand athletes. Managing these injuries can be challenging especially in overhead athletes who require a degree of physiologic laxity to preserve throwing mechanics. Although various treatment methods have previously been described, literature addressing these injuries in overhead throwers remains limited. We describe a technique in which a manually tensionable knotted anchor is placed at the 6:30 position, reducing the chondrolabral complex with an avoidance of overtensioning the tissue to preserve throwing mechanics. This approach provides stable fixation and anatomic reduction of the chondrolabral tissue while minimizing the risk of overconstraint and can be considered a reliable option for treating these lesions in overhead athletes.

## Graphical abstract

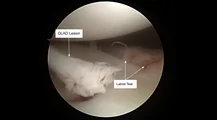

**VIDEO 1:** A standard diagnostic arthroscopy was performed through the standard posterior portal. We established our anterior portals, 1 high and 1 low, and visualized our glenolabral articular disruption lesion. We ensure that we can reach our glenolabral articular disruption with our probe through both of the anterior portals and begin debriding the labral tear as well as the glenolabral articular disruption with a 4.0‐mm shaver. A 15° elevator was used to mobilize the anterior and inferior labral tear. Subsequently, a rasp and a burr were used to prepare the glenoid to stimulate a bleeding surface. A 1.8‐mm all‐suture knotted anchor was placed at the 6:30 position in standard fashion which was used to ensure we can manually tension the chondrolabral flap as the patient is an overhead athlete and we want to avoid overtensioning. A curved suture passer with a nitinol wire was then passed through the anterior band of the inferior glenohumeral ligament underneath the chondrolabral flap. The tissue was then manually tensioned to achieve a reduction of the chondrolabral flap to the glenoid. Moving more lateral, we began placing 1.8 mm all‐suture knotless anchors at the 7:00, 9:30, and 11:00 positions incorporating capsulolabral tissue through our anterior inferior portal. Care was taken to avoid overtensioning the labrum in this patient as he is an overhead athlete and has plans to return to sport. Video content can be viewed at https://doi.org/10.1002/atn2.70294.

Glenolabral articular disruption (GLAD) lesions, in which the glenoid articular cartilage is disrupted, are rare post‐traumatic sequela after shoulder dislocation, present in 1.5 to 2.9% of all shoulder dislocations. Atypical lesions (including GLAD/anterior labral periosteal sleeve avulsions) are thought to be much more prevalent in younger patients, as 41.8% of those aged 15 years and younger were described to have atypical lesions, compared with 17.1% aged 15 years and older^1^ in a 2023 study. GLAD lesions have been shown to have higher rates of failure and recurrent instability than other labral injuries,^2^ although other authors have not shown a difference in long‐term clinical outcomes.^3^ Our purpose is to show a straightforward technique for addressing such lesions with anatomic reduction of capsule, labrum, and avulsed cartilage.

## SURGICAL TECHNIQUE

An examination under anesthesia is performed initially, and the gross stability of the shoulder is assessed. The patient is then placed into the lateral decubitus position, as is the author's preference during instability cases. The surgical extremity is prepped and draped in the usual sterile fashion. A standard posterior portal is established, and a diagnostic arthroscope is performed with a 4.0‐mm 30° arthroscope. During diagnostic arthroscopy, the labral tear is evaluated (Video 1). We visualize the large tear and its extension into the inferior capsule. The GLAD lesion extends anteriorly and inferiorly (shown in Figure 1), with a separation between a large flap of cartilage and the avulsed labrum. A nonengaging Hill‐Sachs lesion is also noted.

**FIGURE 1:**
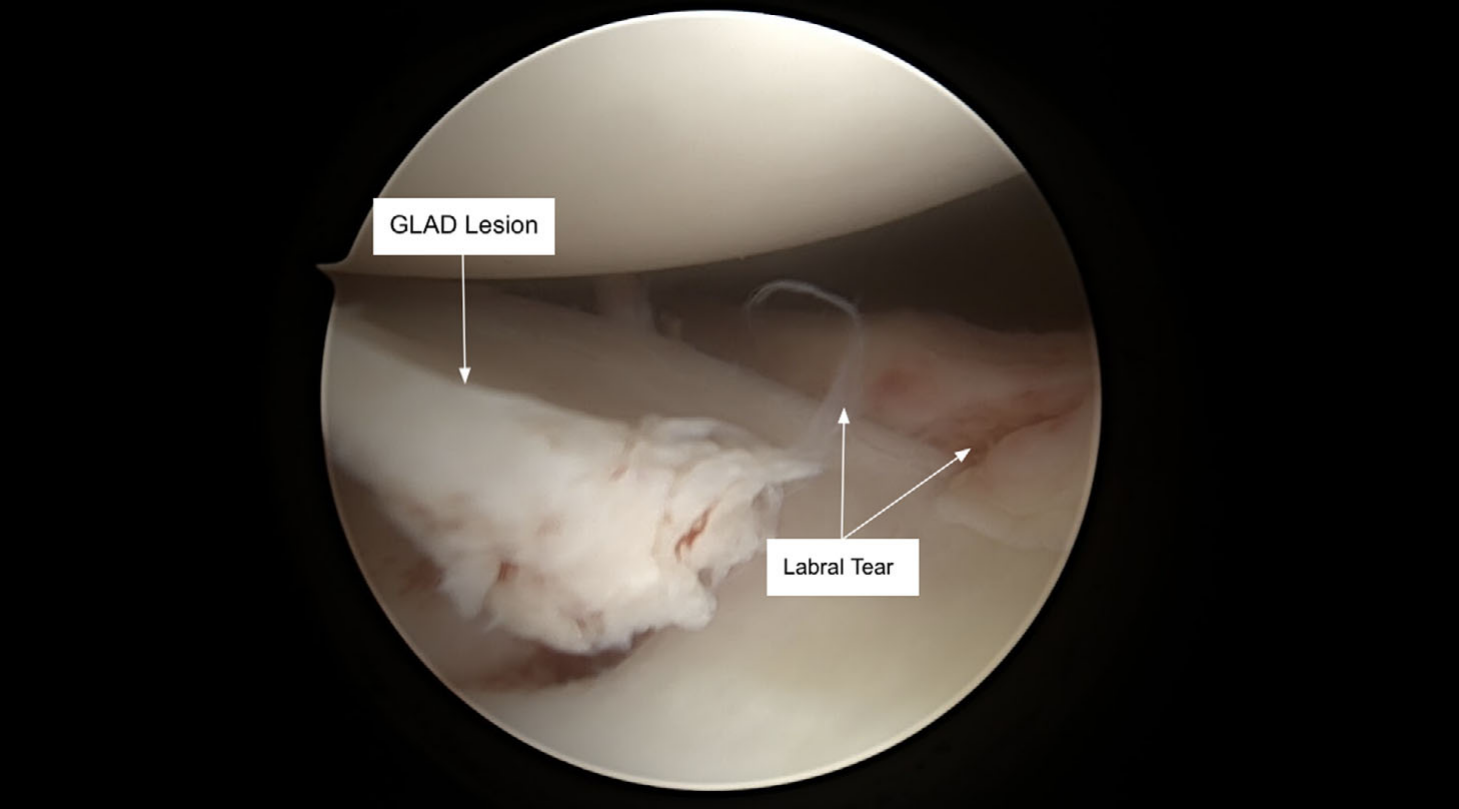
Diagnostic arthroscopy viewing the GLAD lesion and Bankart lesion through the posterior portal. (GLAD, glenolabral articular disruption.)

The anterosuperior and anteroinferior portals are now established through the rotator interval ensuring access to the inferior labrum with a probe. We mobilize the cartilage defect and debride the edges. The labrum is mobilized and can be provisionally reduced to its anatomic position. The glenoid is prepared with a rasp and a burr. A chondroplasty is performed using a 4.0‐mm shaver to obtain clean edges of tissue. The labrum is then mobilized using a 15° elevator. Once stable edges are achieved, a grasper is used to reduce the chondrolabral flap to its anatomic position. Using the anteroinferior portal, a double‐loaded knotted suture anchor (Lupine, DePuy, Raynham, MA) is placed at the 6:30 position, as shown in Figure 2. A 45° suture shuttling device is fed under the labrum and chondral flaps, and the sutures are fed through to reduce this chondrolabral flap. A double‐loaded anchor can be used to grab a broad portion of the tissue to more securely reduce the chondrolabral flap if required. A portion of the anterior band of the inferior glenohumeral ligament is incorporated with the inferior surface of the chondrolabral tissue. This is then tensioned manually with arthroscopic knots, as shown in Figure 3. Care is taken to not overtension this construct, ultimately resulting in a stable inferior capsular shift. Additional knotless 1.8‐mm anchors (Arthrex FiberTak, Naples, FL) are then placed at the 7:00, 9:30, and 11:00 positions while incorporating the capsule and labral tissue, anatomically reducing the labrum in a standard Bankart repair fashion, as shown in Figures 4 and 5. The posterior labrum is then reevaluated to determine the need for further fixation. An additional knotless anchor is placed at the 5:30 position, and the posterior edge of the chondrolabral flap with the posterior band of the inferior glenohumeral ligament is incorporated. A delaminated edge of the GLAD lesion is further debrided at this point to maintain a smooth glenoid face. We perform a synovectomy to debride any hemosiderin synovitis and then irrigate the shoulder copiously. The patient is placed into an ultrasling and instructed to maintain the sling at all times, even during sleep, for the first 4 weeks, only coming out for pendulums and elbow range of motion. He will start therapy at 6 weeks and begin active strengthening 8 to 12 weeks postoperatively.

**FIGURE 2:**
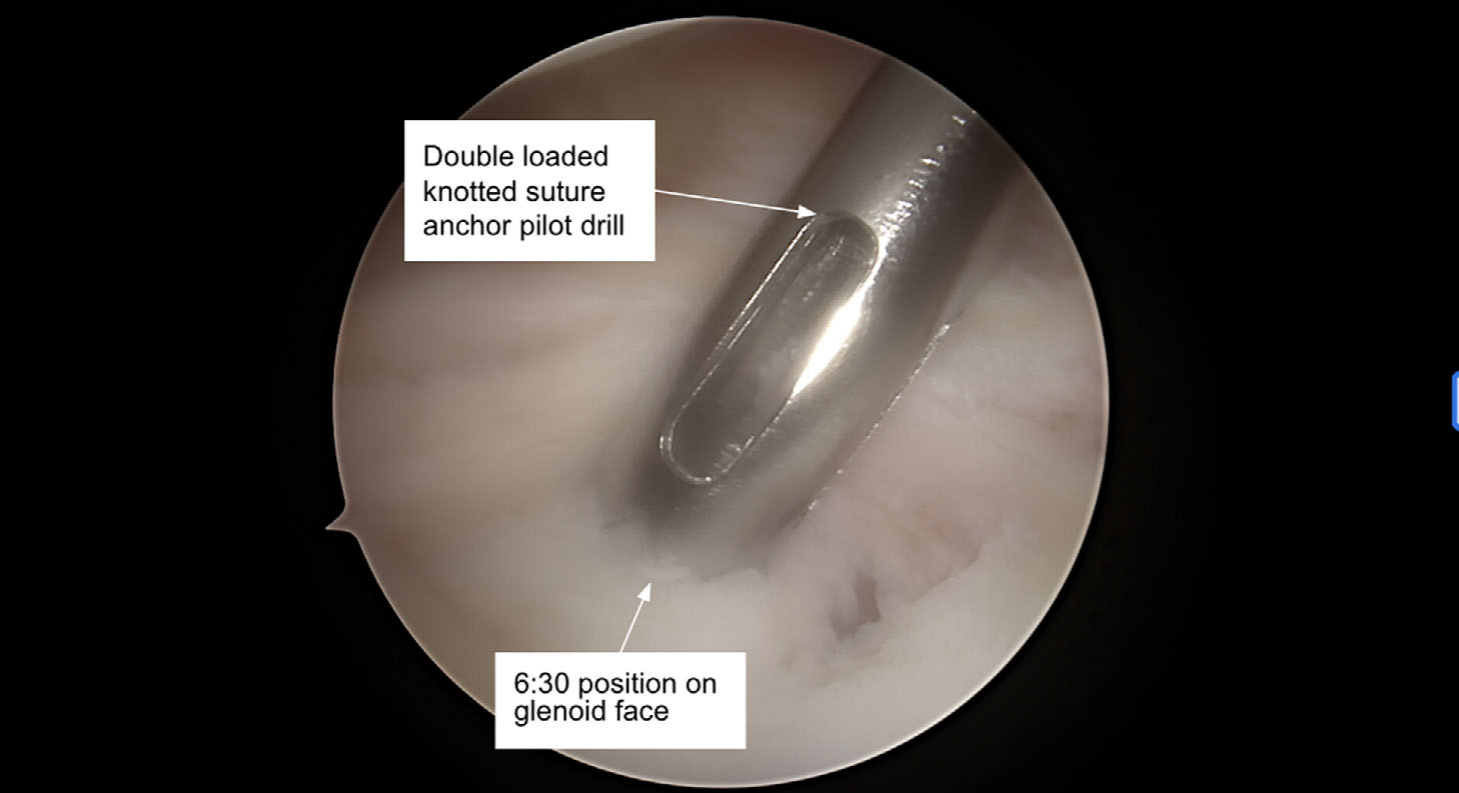
Viewing from the posterior portal as we place our pilot hole for the first anchor near the GLAD lesion at the 6:30 position. This is placed on the margin of the GLAD lesion. (GLAD, glenolabral articular disruption.)

**FIGURE 3:**
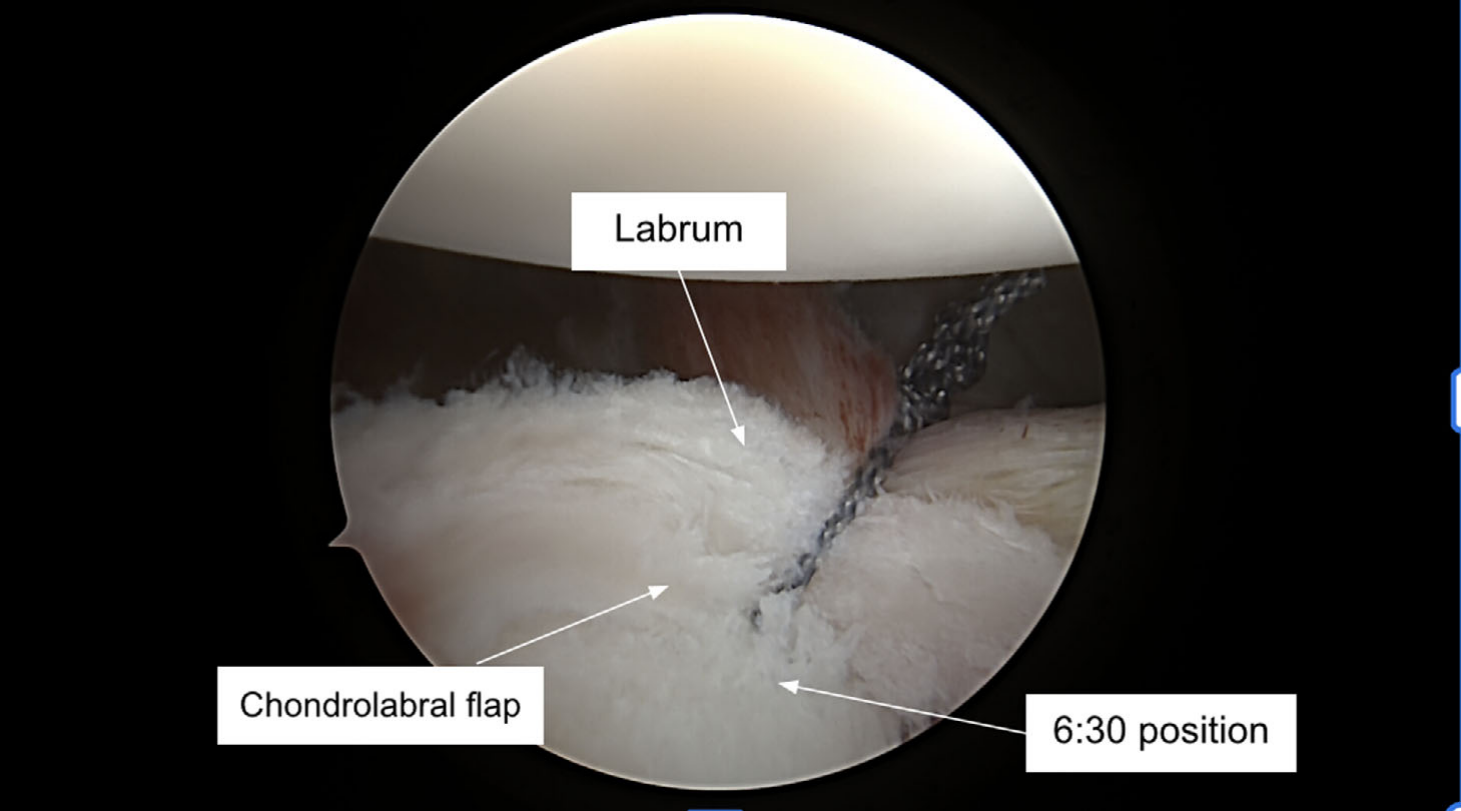
Manually tensioned double‐loaded suture anchor placed at the 6:30 position, reducing the GLAD lesion while incorporating the Bankart tear. Viewing from the posterior portal. (GLAD, glenolabral articular disruption.)

**FIGURE 4:**
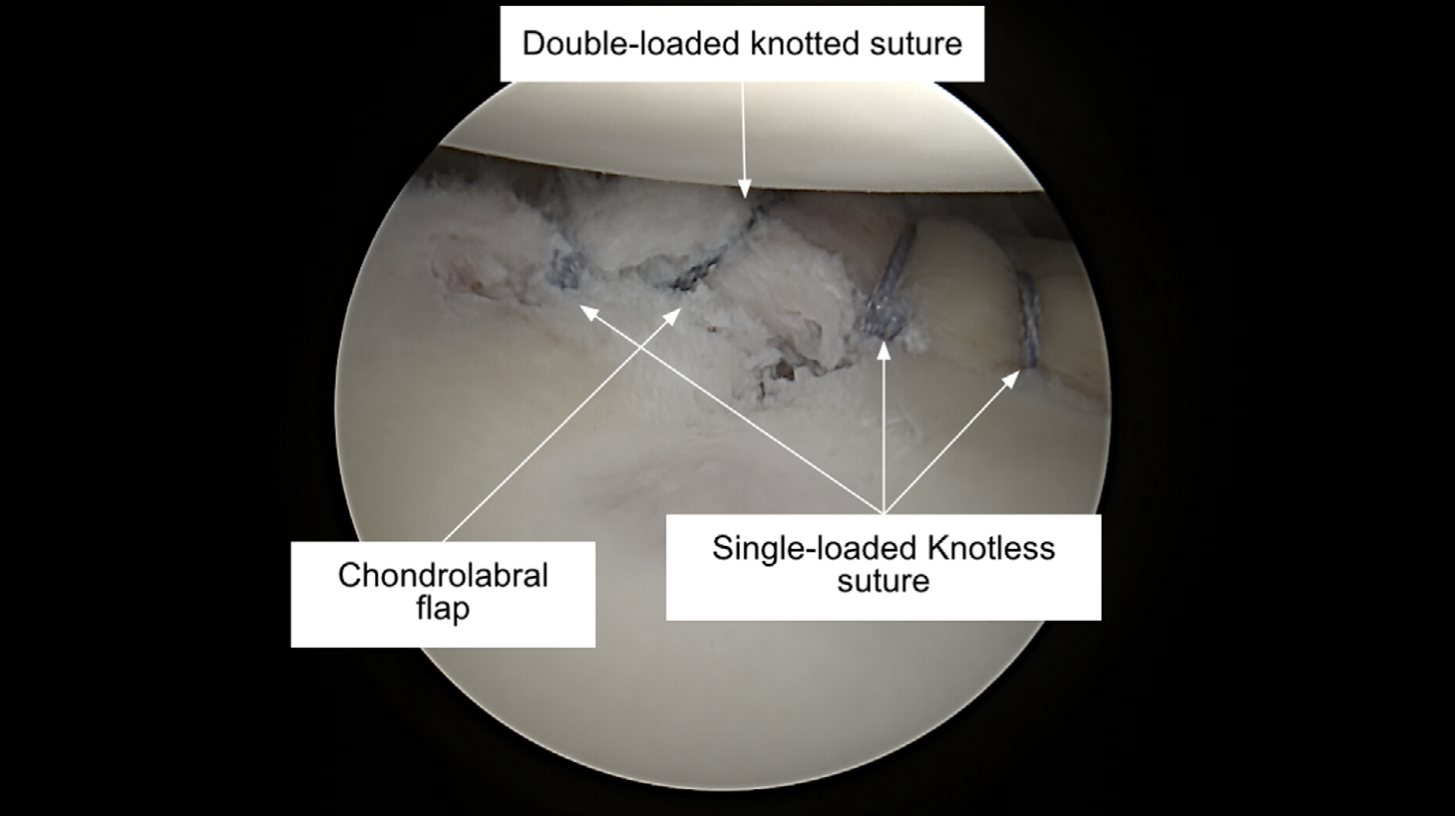
Viewing our repair of the labrum and GLAD lesion from the posterior portal. (GLAD, glenolabral articular disruption.)

**FIGURE 5:**
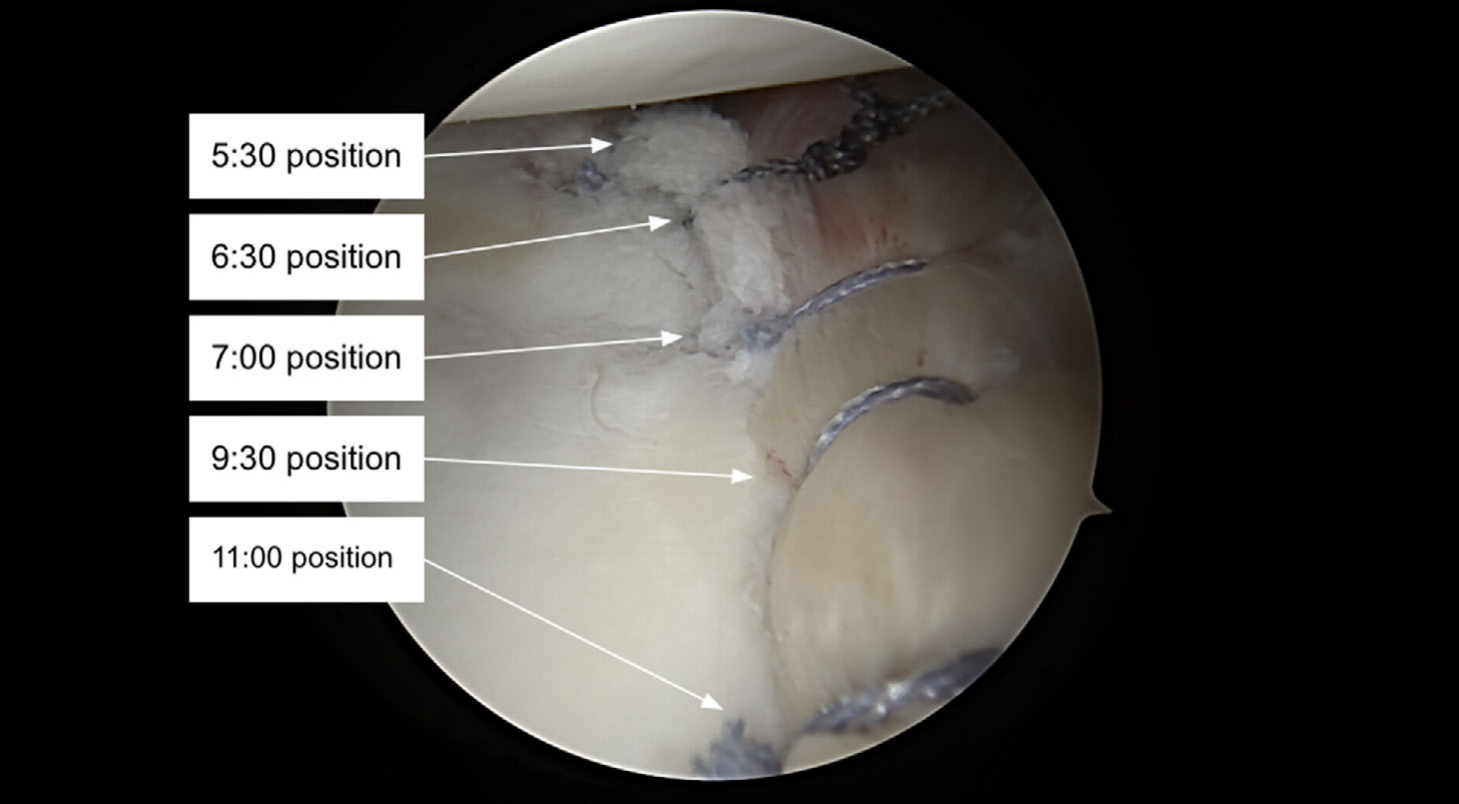
Viewing our completed repair from the posterior portal with anchors at the 5:30, 6:30, 7:00, 9:30, and 11:00 positions.

## DISCUSSION

Glenoid cartilage damage in a GLAD lesion can affect glenoid concavity and contribute to recurrent instability and fixation failure.^2^ Surgical techniques regarding these lesions depend on the extent of injury and can range from debridement and microfracture to stable fixation with various suture anchors or suture bridges.

Because of the rarity of these injuries, a gold standard treatment protocol has yet to emerge. Originally identified in 1993, Neviaser et al. recommended debridement with chondroplasty to avoid mechanical symptoms within the shoulder.^3^ Page et al. in 2010 further suggested suture anchor repair with incorporation of the articular cartilage to prevent recurrent instability from a formerly termed glenoid labral articular flap injury.^4^ Davey et al. described a technique in which the labrum was advanced onto the GLAD lesion, restoring anterior glenoid anatomy and concavity, thereby restoring stability of the glenohumeral joint.^5^ Treatment options continue to vary depending on patient‐specific goals and surgeon experience with GLAD lesions, but fixation or labral advancement tends to be a common approach to managing these injuries.

Our technique included a knotted tensionable 1.8‐mm double‐loaded anchor at the 6:30 position which incorporated the chondrolabral tissue and the disrupted anterior band of the inferior glenohumeral ligament, as discussed in Tables 1 and 2. By employing a knotted all‐suture anchor at this position, we provided stability of the chondrolabral flap and minimized the risk of capsular overtightening. A technique published recently in *Arthroscopy Techniques* highlighted the use of suture‐tape to form a suture‐bridge to reduce the capsulolabral tissue.^6^ We opted to use suture rather than tape for fixation at this location to avoid the risk of tape abrasion of the glenoid cartilage. The Bankart lesion was then repaired using multiple 1.8‐mm FiberTak anchors in a standard fashion around the face of the glenoid incorporating the capsulolabral tissue, as shown in Figures 4 and 5. A remplissage was deliberately avoided to preserve postoperative external rotation, as discussed in Table 1.

**TABLE 1:** Surgical Pearls

**Surgical Pearls**
Adequate debridement and mobilization of the labrum are key to ensure the ability to shift the capsulolabral flap onto the face of the glenoid to incorporate into the chondrolabral defect
Using a double‐loaded suture anchor at the 6:30 position provides a broad‐based fixation construct in a biomechanically advantageous position
Manually tensioning sutures using arthroscopic knots can avoid overtensioning soft tissue in patients who require extreme range of motion

**TABLE 2:** Surgical Pitfalls

**Surgical Pitfalls**
Placing anterior arthroscopic portals too high or too low and not ensuring access to the inferior portion of the glenoid
Undertreating the chondrolabral flap and leaving exposed glenoid cartilage can become a pain generator
Leaving knots or suture anchors proud on the glenoid face which can lead to secondary abrasion and persistent symptoms

Limitations of our technique include the potential for persistent postoperative laxity given that a remplissage was avoided, as well as a theoretical risk of anchor pullout because of its placement in close proximity to the articular defect. Including the chondrolabral flap in fixation has a theoretical additional risk of failure because of the potential for chondrocyte death, thereby loosening the construct.

In summary, this arthroscopic fixation method of a GLAD provides stable reduction of the chondrolabral flap while minimizing the risk of overtightening the shoulder joint, which is advantageous for overhead athletes. Although long‐term outcomes remain to be studied, this technique is a reproducible option for managing these challenging lesions.

## DISCLOSURES

The author (S.K.B.) declares the following financial interests/personal relationships which may be considered as potential competing interests: S.K.B. royalties or licenses—Shoulder Innovations, Atreon, and Trice Medical; consulting fees—Johnson and Johnson, Smith & Nephew, Shoulder Innovations, Pacira, BD, Conmed, and Brinter; payment or honoraria for lectures, presentations, speakers bureaus, manuscript writing, or educational events—Johnson and Johnson, Smith and Nephew, Shoulder Innovations, Pacira, BD, Conmed, and Brinter; patents planned, issued, or pending—Ocean Orthopedics; participation on a Data Safety Monitoring Board or Advisory Board—Kaliber AI, Johnson and Johnson, SutureTech, Sparta, Brinter, and Precision OS; leadership or fiduciary role in other board, society, committee, or advocacy group, paid or unpaid—Chair—AAOS OVT Committee and Membership Committee—ASES; stock or stock options—Kaliber AI, Atreon, SutureTech, Sparta, Brinter, Ocean Orthopedics, and Precision OS; and other financial or nonfinancial interests—Editorial Board—JSES and Editorial Board—JOEI. The other authors (N.F., N.Z., D.B.) declare that they have no known competing financial interests or personal relationships that could have appeared to influence the work reported in this paper.
